# Transcriptome-Wide Profile of 25-Hydroxyvitamin D_3_ in Primary Immune Cells from Human Peripheral Blood

**DOI:** 10.3390/nu13114100

**Published:** 2021-11-16

**Authors:** Andrea Hanel, Igor Bendik, Carsten Carlberg

**Affiliations:** 1 School of Medicine, Institute of Biomedicine, University of Eastern Finland, 70211 Kuopio, Finland; andrea.hanel@uef.fi; 2 DSM Nutritional Products Ltd., Human Nutrition and Health, 4303 Kaiseraugst, Switzerland; igor.bendik@dsm.com

**Keywords:** Vitamin D_3_, 25-hydroxyvitamin D_3_, 1α,25-dihydroxyvitamin D_3_, transcriptome, PBMCs, vitamin D target genes

## Abstract

Vitamin D_3_ is an essential micronutrient mediating pleiotropic effects in multiple tissues and cell types via its metabolite 1α,25-dihydroxyvitamin D_3_ (1,25(OH)_2_D_3_), which activates the transcription factor vitamin D receptor. In this study, we used peripheral blood mononuclear cells (PBMCs) obtained from five healthy adults and investigated transcriptome-wide, whether the precursor of 1,25(OH)_2_D_3_, 25-hydroxyvitamin D_3_ (25(OH)D_3_), has gene regulatory potential on its own. Applying thresholds of >2 in fold change of gene expression and <0.05 as a false discovery rate, in this ex vivo approach the maximal physiological concentration of 25(OH)D_3_ (250 nM (nmol/L)) none of the study participants had a significant effect on their PBMC transcriptome. In contrast, 1000 and 10,000 nM 25(OH)D_3_ regulated 398 and 477 genes, respectively, which is comparable to the 625 genes responding to 10 nM 1,25(OH)_2_D_3_. The majority of these genes displayed specificity to the tested individuals, but not to the vitamin D metabolite. Interestingly, the genes *MYLIP* (myosin regulatory light chain interacting protein) and *ABCG1* (ATP binding cassette subfamily G member 1) showed to be specific targets of 10,000 nM 25(OH)D_3_. In conclusion, 100- and 1000-fold higher 25(OH)D_3_ concentrations than the reference 10 nM 1,25(OH)_2_D_3_ are able to affect the transcriptome of PBMCs with a profile comparable to that of 1,25(OH)_2_D_3_.

## 1. Introduction

When unprotected skin is exhibited to UV-B from sunlight, 7-dehydrocholesterol can convert non-enzymatically to vitamin D_3_ [1]. However, in winter months at latitudes above 40° UV-B, radiation is insufficient for endogenous production of vitamin D_3_ [2]. Under these conditions, the molecule is a real vitamin, which needs to be taken up, in order to keep the vitamin D status at levels that are acceptable for maintaining its disease protective function. The vitamin D status is traditionally determined via the serum levels of the most stable vitamin D_3_ metabolite, 25(OH)D_3_ [3]. Levels of 25(OH)D_3_ below 50 nM (20 ng/mL) are linked to musculoskeletal disorders, such as rickets in children as well as to osteomalacia and fractures in adults [4]. In addition, vitamin D insufficiency is associated with a number of immunological disorders, such as multiple sclerosis [5], rheumatoid arthritis [6], inflammatory bowel disease [7], type I diabetes [8], and increases the risk for severe consequences from infections with mycobacterium tuberculosis [9], influenza virus or severe acute respiratory syndrome coronavirus type 2 (SARS-CoV-2) [10,11]. In order to avoid these risks, the vitamin D status is recommended to be in the order of 100 nM 25(OH)D_3_, i.e., 40 ng/mL [12].

The biologically most active vitamin D_3_ metabolite, 1,25(OH)_2_D_3_, is the only naturally high affinity ligand of the vitamin D receptor (VDR) [13]. VDR is a member of the nuclear receptor superfamily [14] and regulates, as transcription factor, a few hundred target genes in many human tissues and cell types [15,16]. Thus, the function of vitamin D_3_ in health and disease is based on the regulation of the transcriptome of VDR expressing tissues [15]. The vitamin D-dependent transcriptome has been investigated in a number of human tissue and cell lines, such as in THP-1 monocytic leukemia cells [17]. The latter model was extended to primary human cells, of which peripheral blood mononuclear cells (PBMCs) are accessible with minimal harm to the donor [18]. For example, 91 common vitamin D target genes were identified in PBMCs from five healthy adults [19]. Interestingly, an even higher number of genes were found to be personal targets to one or more participants, which fits with our observation that individuals differ largely in their transcriptional response to vitamin D_3_ supplementation [20], i.e., they have a personal vitamin D response index [21].

The enzyme 25(OH)D_3_-1α-hydroxylase is encoded by the gene *CYP27B1* (cytochrome P450 family 27 subfamily B member 1) and converts 25(OH)D_3_ into 1,25(OH)_2_D_3_ [22]. The *CYP27B1* gene is primarily expressed in kidneys, but it is also found in keratinocytes and immune cells, i.e., 1,25(OH)_2_D_3_ is not only produced for endocrine but is also produced for para- and autocrine purposes [23]. In serum vitamin D, metabolites are mainly bound to the serum glycoprotein GC (GC vitamin D binding protein) [24], i.e., both 25(OH)D_3_ and 1,25(OH)_2_D_3_ are mainly protein-bound and only a very small fraction circulates as “free” molecules [22]. 1,25(OH)_2_D_3_ binds with high affinity (K_D_ = 0.1 nM) to the VDR [25], while the affinity of the receptor for 25(OH)D_3_ is 100- to 1000-fold lower [26,27]. However, 25(OH)D_3_ serum levels (50–250 nM) are some 1000-fold higher than that of 1,25(OH)_2_D_3_ (0.05–0.15 nM) [28]. Moreover, molecular dynamics simulations demonstrated that 1,25(OH)_2_D_3_ and 25(OH)D_3_ take the same agonistic conformation within the VDR ligand-binding pocket [29]. This suggests that 25(OH)D_3_ has the potential to act as an agonistic VDR ligand.

In this study, we used PBMCs from healthy individuals participating in the VitDHiD intervention trial (NCT03537027) and investigated in a transcriptome-wide approach the gene regulatory potential of 25(OH)D_3_ in reference to 10 nM 1,25(OH)_2_D_3_.

## 2. Materials and Methods

### 2.1. Sample Collection

Blood samples were collected after overnight (12 h) fasting from five healthy males (individuals numbered 05, 09, 12, 13 and 14, aged 24–54, body mass index 23.0–25.6, vitamin D status 61–118 nM) that participated in the VitDHiD trial [19].

### 2.2. PBMC Isolation and Stimulation

PBMCs were isolated within one hour after collecting 20 mL peripheral blood using Vacutainer CPT Cell Preparation Tubes with sodium citrate (Becton Dickinson) according to manufacturer’s instructions. After washing with phosphate-buffered saline the PBMCs were either stored in liquid nitrogen until use or immediately grown at a density of 0.5 million/mL in 5 mL RPMI 1640 medium supplemented with 10% charcoal-depleted fetal calf serum, 2 mM L-glutamine, 0.1 mg/mL streptomycin and 100 U/mL penicillin at 37 °C in a humidified 95% air/5% CO_2_ incubator. In the first experimental series (S1), freshly isolated PBMCs of all five individuals were exposed for 24 h to either solvent (0.1% EtOH), 250 nM vitamin D_3_ (Sigma–Aldrich, St. Louis, MO, USA), 250 nM 25(OH)D_3_ (Sigma–Aldrich, St. Louis, MO, USA) or 10 nM 1,25(OH)_2_D_3_ (Sigma-Aldrich), while in the second series (S2) PBMCs of individuals numbered 05 and 12 were thawed and together with freshly isolated cells from number 14 stimulated for 24 h with either solvent (0.1% EtOH), 100, 1000 or 10,000 nM 25(OH)D_3_. All experiments were performed for each individual’s cells separately in three repeats.

### 2.3. RNA-Seq Analysis

Total RNA was isolated using the High Pure RNA Isolation Kit (Roche) according to manufacturer’s instructions. RNA quality was assessed on an Agilent 2100 Bioanalyzer system (RNA integrity number ≥ 8). rRNA depletion and cDNA library preparation were performed using the New England Biolabs kits NEBNext rRNA Depletion, NEBNext Ultra II Directional RNA Library Prep for Illumina and NEBNext Multiplex Oligos for Illumina (Index Primers Sets 1 and 2) according to manufacturer’s protocols. RNA-seq libraries went through quality control on an Agilent 2100 Bioanalyzer and were sequenced on a NextSeq 500 system (Illumina) at 75 bp read length using standard protocols at the Gene Core facility of the EMBL (Heidelberg, Germany).

The single-end, reverse-stranded cDNA sequence reads were aligned to the reference genome (version GRCh38) and Ensembl annotation (version 103) using the default settings of the nf-core/rnaseq STAR-Salmon pipeline (version 3.0) [30]. The proportions of mapped and unmapped reads are listed in Figure S1. Ensembl gene identifiers were annotated with gene symbol, description, genomic location and biotype by accessing the Ensembl database (version 103) via the R package BiomaRt (version 2.46.0) [31]. Gene identifiers missing external gene name annotation, genomic location or being mitochondrially encoded were removed from the datasets. When a gene name appeared more than once, the entry with the highest average gene counts was kept.

Differential gene expression analysis was computed in R (version 4.0.2) in the CentOS 7 Linux operating system using the tool EdgeR (version 3.21.1) [32]. For inter-individual transcriptome comparisons, the expression profiles of all 59,372 annotated genes were normalized for differences in library size to counts per million (CPM) and then trimmed mean of M-value normalization was applied, in order to eliminate composition bias between the libraries. The underlying data structure was explored via the dimensionality reduction method multidimensional scaling (MDS) using protein coding genes, in order to visualize relative similarities between samples and detect possible batch effects (Figure S2). MDS was computed via EdgeR’s function plotMDS(), in which distances approximate the typical log2 fold change (FC) between the samples. This distance was calculated as the root mean square deviation (Euclidean distance) of the largest 500 log2FCs between a given pair of samples, i.e., for each pair a different set of top genes was selected. The inspection of the plots showed that samples clustered primarily by treatment and individual, indicating that personal background is a main contributor of variation to the observed gene expression differences (Figure S2). In order to attenuate this confounding effect, we performed the statistical test on each individual’s dataset separately, i.e., the parameters of the negative binomial distribution were estimated from each individual’s transcriptomes. In addition, we reduced our analysis to the 19,908 protein coding genes to mitigate transcriptional noise potentially introduced by non-coding genes.

The gene-wise statistical test for differential expression was computed using the generalized linear model quasi-likelihood pipeline [33]. Genes with very low expression were filtered out by applying the function FilterByExpr(), in order to mitigate the multiple testing problem and to not interfere with the statistical approximations of the EdgeR pipeline. This requirement was fulfilled by 13,284 (number 05), 12,742 (number 09), 13,337 (number 12), 12,530 (number 13) and 13,140 (number 14) genes. After filtering, library sizes were recomputed and trimmed mean of M-value normalization was applied. Trended negative binomial dispersion estimate was calculated using the method CoxReid profile-adjusted likelihood and together with empirical Bayes-moderated quasi-likelihood gene-wise dispersion estimates used for generalized linear model fitting. The empirical Bayes shrinkage was robustified against outlier dispersions as recommended [33]. The glmTreat approach was used to test for differential expression relative to FC > 2 and also for comparison with thresholds of 1.5 and 1.1. Genes with a Benjamini–Hochberg corrected *p*-value, i.e., false discovery rate (FDR) adjusted *p*-value, <0.05 were considered as significant vitamin D targets. Targets with a FC close to infinity (due to expression values close to 0) were excluded from further analysis. Mean-difference (MA) plots were generated with vizzy (version 1.0.0), in order to display the expression profile of each of the 24 comparisons (Figure S3).

Hierarchical clustering was performed using Spearman’s correlation distance on standardized log2FC values of 99 common target genes and visualized in a heatmap via ComplexHeatmap (version 2.2.0). Pathway analysis based on differentially expressed genes was conducted via Enrichr [34,35] using the Kyoto Encyclopedia of Genes and Genomes (KEGG) 2021 human pathways [36] and confirmed with the Signaling Pathway Impact Analysis (SPIA) algorithm [37] implemented in the R package SPIA (version 2.38.0). SPIA is a topology-aware pathway analysis method that considers interactions and dependencies between genes [38]. The analysis was carried out with the setting nB = 2000 on Entrez ID annotated vitamin D target genes (via org.Hs.eg.db (version 3.10.0)) and the mean log2FC of the participants using KEGG 2020 human pathways (release 95.0).

## 3. Results

### 3.1. Impact of Physiological Concentrations of 25(OH)D_3_ on the Transcriptome of PBMCs

**Figure 1:**
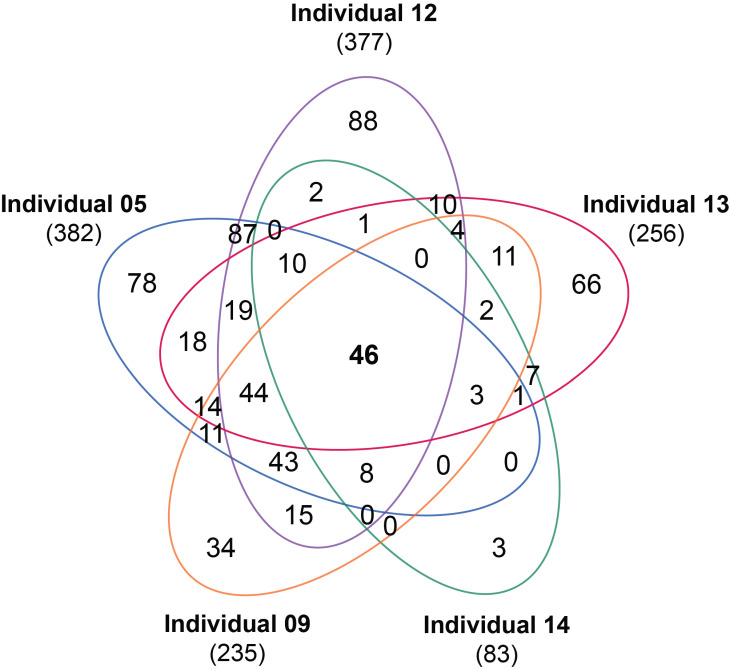
Common and personal target genes of 1,25(OH)_2_D_3_ in PBMCs. PBMCs isolated from five individuals were treated ex vivo in triplicate with 10 nM 1,25(OH)_2_D_3_. Statistical analysis identified 83 to 382 significantly regulated genes per individual (FC > 2, FDR < 0.05) and a Venn diagram displays the overlap of the respective five sets of vitamin D targets.

PBMCs from five healthy male adults were obtained in context of the vitamin D intervention trial VitDHiD (NCT03537027), which aimed to investigate the gene regulatory impact of 25(OH)D_3_. Immediately after isolation, the cells were stimulated for 24 h with either solvent, 250 nM vitamin D_3_, 250 nM 25(OH)D_3_ or 10 nM 1,25(OH)_2_D_3_ and RNA-seq analysis was performed. When applying this triplicate ex vivo approach, the rather strict thresholds of FC > 2 and FDR < 0.05, we did not observe any target genes of 250 nM vitamin D_3_ or 250 nM 25(OH)D_3_ in PBMCs of none of the five tested individuals (Table 1). As a reference, under the same conditions we found 382, 377, 256, 235 and 83 targets of 1,25(OH)_2_D_3_ in PBMCs of individuals numbered 05, 12, 13, 09 and 14, respectively. In sum of all individuals, 625 different genes were identified for 1,25(OH)_2_D_3_ targets, the majority (67.7%) of which were down-regulated (Table S1). However, only 46 (7.4% of all) of these genes were in common for all five individuals, an additional 65 genes (10.4%) were found in four, 84 (13.4%) in three, 161 (25.8%) in two and 269 (43.0%) were personal to one of the study participants (Figure 1). For comparison, when we reduced the FC threshold to 1.5 or even 1.1, the number of genes regulated by 1,25(OH)_2_D_3_ drastically increased, but 250 nM vitamin D_3_ or 250 nM 25(OH)D_3_ still did not significantly regulate any gene (Table S2).

In summary, our ex vivo approach demonstrated that high physiological concentrations (250 nM) of neither vitamin D_3_ nor 25(OH)D_3_ resulted in any significant changes of the PBMCs transcriptome of healthy adults. In comparison, a 25-fold lower concentration of 1,25(OH)_2_D_3_, also representing a very high concentration for this vitamin D metabolite, modulated the transcriptome of 625 genes in total, although the majority of them in an individual-specific fashion.

### 3.2. Effects of High Concentrations of 25(OH)D_3_ on the PBMC Transcriptome

Since 25(OH)D_3_ levels, which are considered to be in the physiological range, had no significant effect on the PBMC transcriptome, we next tested stochiometric concentrations to the reference 10 nM 1,25(OH)_2_D_3_. Assuming a 100–1000 fold lower affinity of VDR to 25(OH)D_3_ than to 1,25(OH)_2_D_3_, these were 1000 and 10,000 nM 25(OH)D_3_. In addition, 100 nM 25(OH)D_3_ served as a further control. The same triplicate ex vivo approach with PBMCs was used (obtained this time only from individuals numbered 05, 12 and 14) and the thresholds FC > 2 and FDR < 0.05 were again applied. As expected on the basis of the experience with 250 nM 25(OH)D_3_, a concentration of 100 nM was not able to significantly modulate the transcriptome of PBMCs of any of the three study participants (Table 2).

However, the high concentrations of 25(OH)D_3_ regulated for each individual nearly as many genes as 1,25(OH)_2_D_3_ did at a concentration of 10 nM. PBMCs of individual number 05 showed the most prominent response to the treatments with 1000 and 10,000 nM 25(OH)D_3_ as well as to 10 nM 1,25(OH)_2_D_3_, which overlapped in 215 genes (Figure S4A). For comparison, PBMCs of individual number 12 (Figure S4B) and individual number 14 (Figure S4C) had 168 and 22 commonly responding genes, respectively.

**Figure 2:**
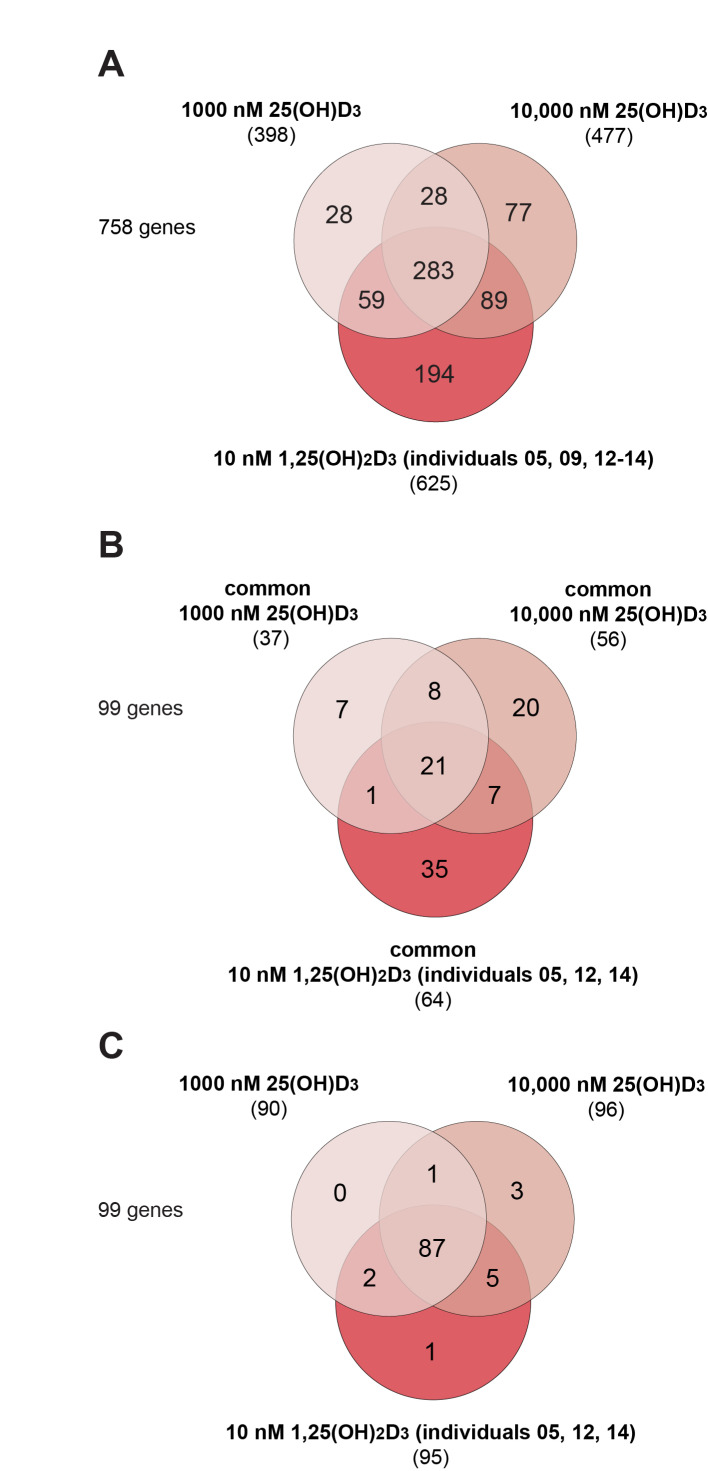
Overview of differential gene expression. Venn diagrams represent the overlap of target genes of 1000 nM 25(OH)D_3_ (identified in individuals numbered 05, 12 and 14), 10,000 nM 25(OH)D_3_ (in individuals numbered 05, 12 and 14) and 10 nM 1,25(OH)_2_D_3_ (identified in all five study subjects) (**A**), the in total 99 common target genes (in individuals numbered 05, 12 and 14) (**B**) or the result of filtering all identified target genes by the common 99 target genes (**C**).

Taking the transcriptome profiles of three individuals together, 398 different genes responded significantly to the concentration of 1000 nM 25(OH)D_3_ (67.1% down-regulated) and even 477 genes were targets of 10,000 nM 25(OH)D_3_ (75.3% down-regulated) (Figure 2A). Both lists overlapped in 311 genes, 283 (91.0%) of which had also been found as targets of 1,25(OH)_2_D_3_ in PBMCs of at least one of the five investigated study participants. However, this also suggests that in at least one individual, 28 genes seem to be specific targets to 1000 nM 25(OH)D_3_, even 77 genes responded only to 10,000 nM 25(OH)D_3_, while 28 genes were regulated by both concentrations of 25(OH)D_3_.

**Figure 3:**
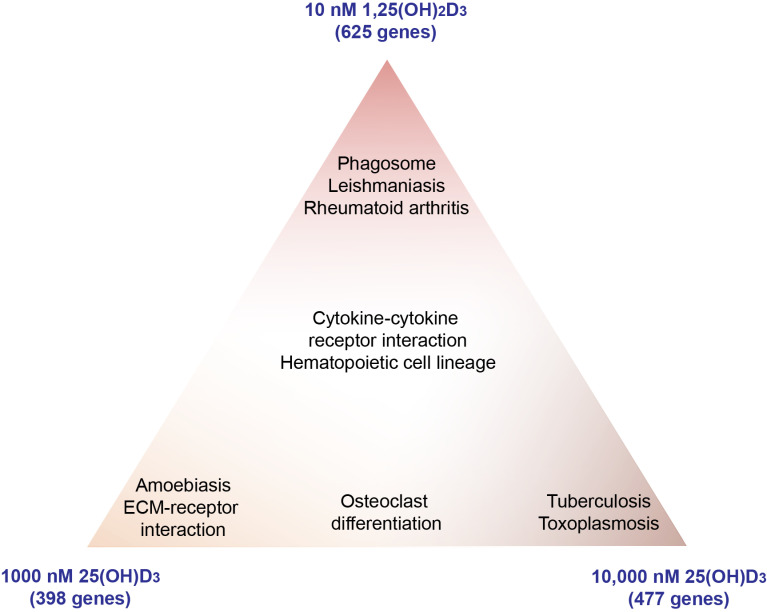
Visualization of the top 5 KEGG pathway overlap after PBMC treatment.

Pathway analysis using the webtool Enrichr with the 398, 477 and 625 target genes of 1000 nM 25(OH)D_3_, 10,000 nM 25(OH)D_3_ and 10 nM 1,25(OH)_2_D_3_, pointed to their top five functions based on KEGG pathways. The treatment with 1000 nM 25(OH)D_3_ associated with “Hematopoietic cell lineage”, “Amoebiasis”, “ECM-receptor interaction”, “Cytokine–cytokine receptor interaction” and “Osteoclast differentiation” (Table S3). A 10-fold higher 25(OH)D_3_ level also resulted in “Cytokine-cytokine receptor interaction”, “Osteoclast differentiation” and “Hematopoietic cell lineage”, and additionally in “Tuberculosis” and “Toxoplasmosis”. The stimulation with 1,25(OH)_2_D_3_ confirmed “Cytokine–cytokine receptor interaction” as well as “Hematopoietic cell lineage” and added “Leishmaniasis”, “Rheumatoid arthritis” and “Phagosome” to the list of the most significant pathways (Figure 3). For comparison, we also performed topology-aware pathway analysis using the SPIA method and verified “Cytokine–cytokine receptor interaction” for all three treatment conditions as well as “ECM-receptor interaction”, “Amoebiasis” and “Osteoclast differentiation” for 1000 nM 25(OH)D_3_, “Osteoclast differentiation”, “Tuberculosis” and “Toxoplasmosis” for 10,000 nM 25(OH)D_3_ and “Leishmaniasis” and “Rheumatoid arthritis” for 1,25(OH)_2_D_3_ (Table S4).

Taken together, 1000 and 10,000 nM of 25(OH)D_3_, but not the physiological concentration of 100 nM, are able to regulate a similar set of genes as 10 nM 1,25(OH)_2_D_3_. Pathway analysis methods agreed on “Cytokine–cytokine receptor interaction” as a common function of all three types of stimulation.

### 3.3. Target Genes Specific to 25(OH)D_3_

The transcriptome-wide response of the three tested individuals to a stimulation with 25(OH)D_3_ was rather divergent, as they overlapped in only 37 target genes at a concentration of 1000 nM (Figure S5A) and in 56 genes at 10,000 nM (Figure S5B). However, these numbers were in the same order as the 64 genes that are common targets of 1,25(OH)_2_D_3_ of individuals numbered 05, 12 and 14 (Figure S5C). From these, a total of 99 common targets and only 21 genes responded in all three individuals to all three types of stimulation, while 7, 20 and 35 genes appeared to be specific to a treatment with 1000 nM 25(OH)D_3_, 10,000 nM 25(OH)D_3_ and 10 nM 1,25(OH)_2_D_3_, respectively (Figure 2B).

The 99 common target genes were used as a filter for the set of 758 genes (Figure 2A and Table S1) responding to at least one treatment, in at least one of the individuals (Figure 2C). This approach suggested that only one gene (*TNFRSF18* (TNF receptor superfamily member 18)) responds specifically to 1,25(OH)_2_D_3_, no gene to 1000 nM 25(OH)D_3_ and three genes (*ABCG1*, *MYLIP* and *CSF1R* (colony stimulating factor 1 receptor)) to 10,000 nM 25(OH)D_3_. Moreover, one gene (*KCNF1* (potassium voltage-gated channel modifier subfamily F member 1)) was a target of both 1000 nM and 10,000 nM 25(OH)D_3_, two genes (*PDPN* (podoplanin) and *TREM1* (triggering receptor expressed on myeloid cells 1)) were common to 1000 nM 25(OH)D_3_ and 1,25(OH)_2_D_3_ as well as five genes (*IL13RA1* (interleukin 13 receptor subunit alpha 1), *HCAR3* (hydroxycarboxylic acid receptor 3), *ARHGEF40* (Rho guanine nucleotide exchange factor 40), *LAD1* (ladinin 1) and *CLEC5A* (C-type lectin domain containing 5A)) that were regulated by both 10,000 nM 25(OH)D_3_ and 1,25(OH)_2_D_3_ (Table S1).

**Figure 4:**
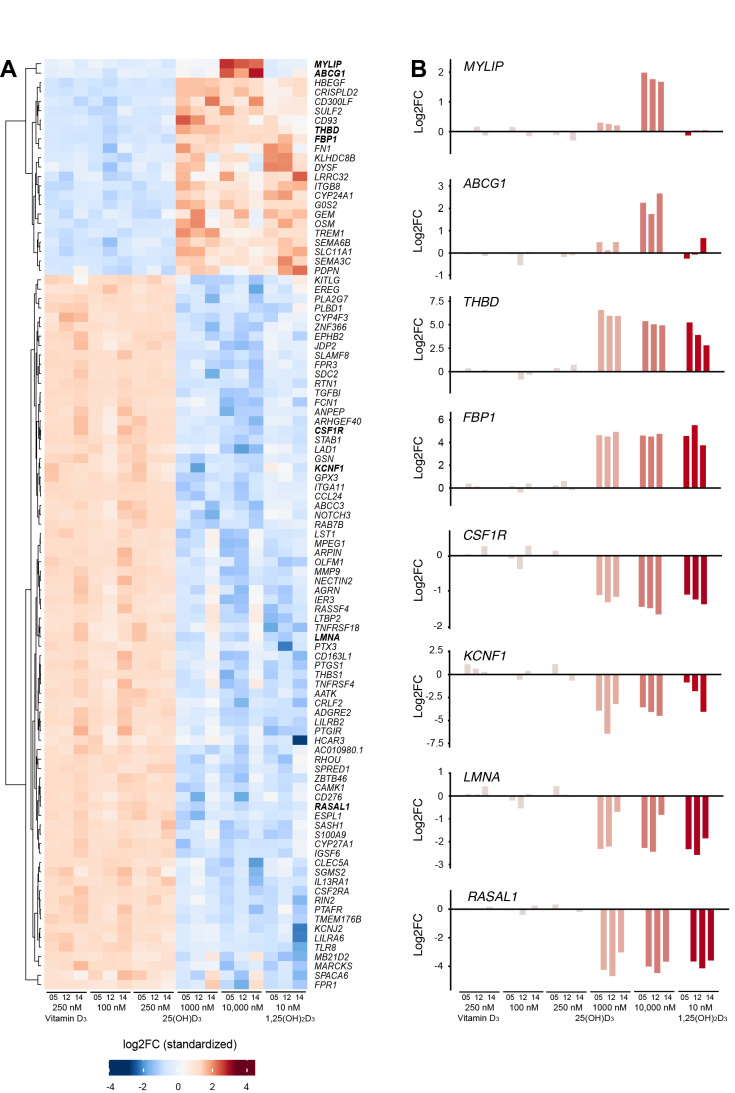
Vitamin D target genes in PBMCs. PBMCs isolated from individuals numbered 05, 12 and 14 were treated in vitro in triplicate with 250 nM vitamin D_3_, 100, 250, 1000 or 10,000 nM 25(OH)D_3_ or 10 nM 1,25(OH)_2_D_3_ (125D). Venn diagram identified 99 common vitamin D target genes (Figure 2B). A heatmap was used for displaying the change of gene expression (standardized log2FC) of the 99 common vitamin D target genes (**A**). Hierarchical clustering was applied on genes to elucidate, in an unsupervised manner, the expression patterns between treatments. Bar charts are used to display the change of expression (log2FC) of the up-regulated vitamin D target genes *MYLIP*, *ABCG1*, *THBD* and *FBP1* as well as of the down-regulated genes *CSF1R*, *KCNF1*, *LMNA* and *RASAL1* (**B**).

An alternative view on the 99 vitamin D target genes was provided by a heatmap using hierarchical clustering (Figure 4A). This map clearly distinguished 23 genes being up-regulated by the vitamin D compounds from 76 down-regulated genes. Moreover, it highlighted with *MYLIP* and *ABCG1* a sub-cluster of genes that were specifically up-regulated by 10,000 nM 25(OH)D_3_. In contrast, the heatmap did not suggest any specificity of the 10 additional genes, which had been indicated by the filtered Venn diagram (Figure 2C). Bar charts emphasized the up-regulated genes *MYLIP* and *ABCG1* as specific targets of 10,000 nM 25(OH)D_3_, while the apparent specific response of the down-regulated genes *CSF1R* and *KCNF1* to 25(OH)D_3_ could not be confirmed (Figure 4B). The well-known up-regulated vitamin D target genes *THBD* (thrombomodulin) [20,39] and *FBP1* (fructose-bisphosphatase 1) [40,41] and the down-regulated genes *LMNA* (lamin A/C) [42] and *RASAL1* (RAS protein activator like 1) [19] served as references. Moreover, the suggested specificity of the up-regulated genes *TREM1* and *PDPN* as well as of the down-regulated genes *ARHGEF40*, *LAD1*, *TNFRSF18*, *HCAR3*, *CLEC5A* and *IL13RA1*, are based on inter-individual variations, since their response profile is similar to *THBD* and *FBP1* or *LMNA* and *RASAL1* (Figure S6).

In summary, from 99 common vitamin D target genes in human PBMCs, only *MYLIP* and *ABCG1* were specifically regulated by 10,000 nM 25(OH)D_3_, while the suggested vitamin D compound or concentration specificity of 10 other genes could not be verified by further inspection.

## 4. Discussion

The aim of this study was to investigate the gene regulatory potential of 25(OH)D_3_ on a transcriptome-wide level. Since highest levels of 25(OH)D_3_ are found in serum, vitamin D responsive cells of the blood are the first and most obvious target of a possible gene regulatory effect of the vitamin D_3_ metabolite. Therefore, we used freshly isolated PBMCs of five healthy individuals as an ex vivo experimental system for testing different concentrations of 25(OH)D_3_ (100, 250, 1000 and 10,000 nM) in relation a standard concentration of 1,25(OH)_2_D_3_ (10 nM). From our own experience [19,43] and the literature [44], we know that the number of regulated genes obtained by transcriptome-wide analysis largely depends on threshold settings, both in minimal changes of expression (FC) as well as on the chosen statistical approach. In this study, we applied the rather rigorous statistical test of glmTreat [32,33] using thresholds of FC > 2 and FDR < 0.05 in order to focus on reliably regulated genes. Nevertheless, under these conditions, a 24 h stimulation with 1,25(OH)_2_D_3_ still resulted in 83 to 382 vitamin D target genes for the five tested individuals. Using this reference, we observed that neither a 25(OH)D_3_ concentration of 100 nM nor 250 nM, which are both within the physiological range, resulted in PBMCs of any of the study participants in a significant regulation of genes. A further reference, 250 nM vitamin D_3_, also did not change the PBMC transcriptome. The finding on the transcriptome profile of physiological concentrations of 25(OH)D_3_ and vitamin D_3_ was not only obtained from counts of genes passing the thresholds but is also obvious from large-scale visualizations, such as MDS, MA plots and a heatmap. Thus, one major result of this study is that in healthy humans, the normal range of 25(OH)D_3_ and vitamin D_3_ serum concentrations may not activate the VDR in PBMCs and change the expression of its target genes. This fits with a transcriptome analysis of muscle biopsies from elderly individuals, where supplementation with 10 µg 25(OH)D_3_ per day had no significant effect on gene expression [45].

With the same confidence as we indicated that physiological concentrations of 25(OH)D_3_ did not change the PBMC transcriptome, we demonstrated that higher concentrations of the vitamin D metabolite, such as 1000 and 10,000 nM, are able to affect gene expression. A level of 1000 nM 25(OH)D_3_ may be reached in vivo by high overdosing of vitamin D_3_ supplementation, such as a daily bolus of 250 µg (10.000 IU) or more, but a level of 10,000 nM can only be obtained in vitro or under short-term special treatment requiring medical supervision. Nevertheless, these high concentrations of 25(OH)D_3_ significantly regulate a comparable number of vitamin D target genes as observed with 10 nM 1,25(OH)_2_D_3_.

This study confirmed our previous observation [19] that there are large inter-individual differences in the number and identity of vitamin D target genes, when PBMCs of different individuals are stimulated with 1,25(OH)_2_D_3_. Comparably, we found that also a treatment with 1000 or 10,000 nM 25(OH)D_3_ results in large inter-individual differences. However, we found 99 common target genes for all three types of PBMC stimulations. The observation of (i) comparable number of target genes in total, (ii) their inter-individual variation and (iii) nearly 100 common targets suggest that 25(OH)D_3_ uses the same mechanism of gene regulation as 1,25(OH)_2_D_3_. Concerning genomic mechanisms of vitamin D signaling [46] the latter conclusion is obvious, since VDR is the exclusive high affinity target of 1,25(OH)_2_D_3_. However, there are a number of non-genomic mechanisms discussed, which may explain particular effects of high concentrations of vitamin D compounds [47].

Under the assumption that target genes of 25(OH)D_3_ primarily act via the VDR, there are two major options: (i) the molecule either directly acts as an agonistic ligand of the nuclear receptor or (ii) sufficient amounts of 25(OH)D_3_ are enzymatically converted to 1,25(OH)_2_D_3_, so that a concentration of the latter > 0.1 nM is reached, which directly activates the receptor. PBMCs of all five tested individuals show a very low but significant expression of the *CYP27B1* gene, so that the second option cannot be excluded. Specific binding of ligands to the ligand-binding pocket within VDR’s ligand-binding domain is achieved via three pairs of polar amino acids interacting with the three OH-groups of 1,25(OH)_2_D_3_; S237 and R274 bind the 1-OH-group, S278 and Y143 the 3-OH-group and H305 and H397 the 25-OH-group [48,49]. Since 25(OH)D_3_ lacks a 1-OH-group, it binds with lower affinity to the VDR. However, 25(OH)D_3_ concentrations of 1000 nM and more should be more than sufficient for effective binding and acting as VDR agonist [26,50]. Nevertheless, even if 25(OH)D_3_ may act as direct VDR ligand, the experience with synthetic vitamin D analogs [51] has shown that there is only one agonistic VDR conformation, i.e., 25(OH)D_3_ and 1,25(OH)_2_D_3_ should activate the same set of genes.

From the 398 targets of 1000 nM 25(OH)D_3_ 85.9% responded also to 1,25(OH)_2_D_3_ in PBMCs of at least one of the five investigated study participants, while for the 477 target genes of 10,000 nM 25(OH)D_3_ the rate was with 78.0% a bit lower. The 99 common targets of 1000 nM 25(OH)D3, 10,000 nM 25(OH)D_3_ and 10 nM 1,25(OH)_2_D_3_ allowed a more focused view and initially suggested that 12 genes display specificity. However, only the genes *MYLIP* and *ABCG1* were shown to be specifically regulated by 10,000 nM 25(OH)D_3_. Interestingly, both genes are involved in cholesterol transport and are known to be regulated by the nuclear receptor LXR (liver X receptor) [52,53,54] but had not been reported as VDR targets. Moreover, vitamin D metabolites, including 25(OH)D_3_, have been shown to activate LXR [55], i.e., the specific regulation of *MYLIP* and *ABCG1* may be mediated rather by LXR than by VDR. Another important regulator of cholesterol are the transcription factors SREBF (sterol regulatory element binding transcription factor) 1 and 2, which are attached to the membrane of the endoplasmic reticulum in their inactive form [56]. The activation of SREBFs is controlled by the sterol-binding protein SCAP (SREBF chaperone), i.e., cholesterol levels control the transcription factors and their target genes [57]. Interestingly, it was shown that 25(OH)D_3_ inhibits the activation of SREBFs via the induction of SCAP degradation [58]. This may be an alternative mechanism explaining the specific up-regulation of the genes *MYLIP* and *ABCG1* by high levels of 25(OH)D_3_. Thus, there are at least two VDR-independent mechanisms that could define 25(OH)D_3_-specific gene regulation.

Finally, some limitations of this study need to be considered. The initial design involved only the comparison of 250 nM 25(OH)D_3_ and 250 nM vitamin D_3_ to 10 nM 1,25(OH)_2_D_3_ where freshly isolated PBMCs from five individuals were used (S1), while experiments with higher 25(OH)D_3_ concentrations were carried out later on thawed cells from individuals numbered 05 and 12 and new freshly isolated cells from individual 14 (S2). Thus, the non-overlapping part of the transcriptomes of 25(OH)D_3_ and 1,25(OH)_2_D_3_ particularly seen with individual number 14, may be attributed to differences between the two batches of cells. Furthermore, since PBMCs are a diverse mixture of myeloid and lymphoid cells, the differences in frequency of different cell populations between individuals and experimental batches also may have contributed to the observed inter- and intra-individual transcriptional differences.

In conclusion, this study demonstrated that physiological concentrations of 25(OH)D_3_ do not have any significant effect on the transcriptome of human PBMCs. At concentrations of 100 and 250 nM 25(OH)D_3,_ we did not observe any gene regulation; while at 1000 and 10,000 nM, the number and identity of target genes was comparable to that of 1,25(OH)_2_D_3_. Intriguingly, specific high concentration effects of 25(OH)D_3_, such as the up-regulation of the genes *MYLIP* and *ABCG1*, may be explained by VDR-independent mechanisms involving either LXR or SREBFs.

## Figures and Tables

**Table 1:** Differentially regulated genes. PBMCs of all five individuals were treated in triplicate for 24 h with the designated vitamin D compounds. RNA-seq analysis was performed and the number of total up- and down-regulated target genes (FC > 2, FDR < 0.05) as well as of all expressed protein coding genes used in the statistical test are indicated.

Individual Number	Treatment	Concentration (nM)	Target Genes Total	Target Genes Up	Target Genes Down	Genes Expressed
05	Vitamin D_3_	250	0	0	0	13,284
05	25(OH)D_3_	250	0	0	0	13,284
05	1,25(OH)_2_D_3_	10	382	122	260	13,284
09	Vitamin D_3_	250	0	0	0	12,742
09	25(OH)D_3_	250	0	0	0	12,742
09	1,25(OH)_2_D_3_	10	235	57	178	12,742
12	Vitamin D_3_	250	0	0	0	13,337
12	25(OH)D_3_	250	0	0	0	13,337
12	1,25(OH)_2_D_3_	10	377	131	246	13,337
13	Vitamin D_3_	250	0	0	0	12,530
13	25(OH)D_3_	250	0	0	0	12,530
13	1,25(OH)_2_D_3_	10	256	53	203	12,530
14	Vitamin D_3_	250	0	0	0	13,140
14	25(OH)D_3_	250	0	0	0	13,140
14	1,25(OH)_2_D_3_	10	83	20	63	13,140

**Table 2:** Gene regulatory potential of 25(OH)D_3_. PBMCs of individuals numbered 05, 12 and 14 were treated in triplicate for 24 h with increasing concentrations of 25(OH)D_3_. RNA-seq analysis was performed and the number of total up- and down-regulated target genes (FC > 2, FDR < 0.05) as well as of all expressed (and for differential expression tested) protein coding genes are indicated.

Individual Number	Treatment	Concentration (nM)	Target Genes Total	Target Genes Up	Target Genes Down	Genes Expressed
05	25(OH)D_3_	100	0	0	0	13,284
05	25(OH)D_3_	1000	332	122	210	13,284
05	25(OH)D_3_	10,000	386	90	296	13,284
12	25(OH)D_3_	100	0	0	0	13,337
12	25(OH)D_3_	1000	265	99	166	13,337
12	25(OH)D_3_	10,000	341	63	278	13,337
14	25(OH)D_3_	100	0	0	0	13,140
14	25(OH)D_3_	1000	47	15	32	13,140
14	25(OH)D_3_	10,000	66	14	52	13,140

## Data Availability

Fastq files of the 96 libraries can be found at Gene Expression Omnibus (GEO) with accession numbers GSE156124 and GSE179225.

## Supplementary Materials

The following are available online at https://www.mdpi.com/article/10.3390/nu13114100/s1, Figure S1: Read alignment, Figure S2: Sample quality assessment via MDS, Figure S3: Global effects of treatment, Figure S4: Individual-specific differential gene expression, Figure S5: Compound-specific differential gene expression, Figure S6: Profile of selected vitamin D target genes, Table S1: The vitamin D-triggered transcriptome of PBMCs, Table S2: Comparison of FC thresholds, Table S3: Significantly impacted pathways analyzed by Enrichr, Table S4: Significantly impacted pathways analyzed by SPIA.
