# Supplementary material for: Transcriptome-Wide Profile of 25-Hydroxyvitamin D3 in Primary Immune Cells from Human Peripheral Blood
Source: Nutrients. 2021 Nov 16;13(11):4100. doi: 10.3390/nu13114100 (PMC8624141; doi:10.3390/nu13114100)
Supplement: Supplementary file 1 [file nutrients-13-04100-s001.zip › nutrients-1405716-supplementary/Supplementary material/Supplementary legends.pdf]

SUPPLEMENTARY TABLE LEGENDS

Table S1: The vitamin D-triggered transcriptome of PBMCs. In experimental series 1 PBMCs of individuals #05, #09, #12, #13 and #14 were treated with solvent (0.1% EtOH), 250 nM vitamin D_3 (D3), 250 nM 25(OH)D_3 (25D) or 10 nM 1,25(OH)$_2$$D_3$ (125D), while in series 2 PBMCs of individuals #05, #12 and #14 were stimulated with solvent, 100, 1000 or 10,000 nM 25(OH)D_3. Treatment was in triplicate for 24 h, RNA was extracted and subjected to RNA-seq analysis. Differentially expressed genes were identified using the statistical test *glmTreat* with the thresholds FC > 2 and FDR < 0.05. For a given treatment condition data are shown only for those genes that passed the threshold. In total 758 genes are listed that at least in one of the five individuals responded to 25(OH)D_3, 1,25(OH)$_2$$D_3$ or both. Tables of complete datasets of all expressed genes are found at GEO with accession number GSE179225.

Table S2. Comparison of FC testing thresholds. In extension of **Table 1** the number of target genes were calculated with glmTreat test using FC thresholds of 1.5 and 1.1. Genes with FDR < 0.05 were counted as target genes.

Table S3: Significantly impacted pathways analyzed by Enrichr. Functional analysis was performed using the webtool Enrichr [34] on vitamin D target genes identified based on 10 nM 1,25(OH)$_2$$D_3$ (all 5 individuals) as well as 1000 and 10,000 nM 25(OH)D_3 (individuals #05, #12 and #14). The top 5 KEGG pathways, sorted by FDR, are listed for each of the three treatments for the FC thresholds > 2, > 1.5 and > 1.1.

Table S4: Significantly impacted pathways analyzed by SPIA. Functional analysis was performed using SPIA algorithm on vitamin D target genes identified based on 10 nM 1,25(OH)$_2$$D_3$ (all 5 individuals) as well as 1000 and 10,000 nM 25(OH)D_3 (individuals #05, #12 and #14) (**Fig. 2A**). The top 5 KEGG pathways, sorted by Bonferroni-adjusted global p-values (pGFWER), are listed for each of the three treatments.

SUPPLEMENTARY FIGURE LEGENDS

Fig. S1: Read alignment. Proportions of unmapped, multi-mapping and uniquely aligned reads per each sample are indicated. S1, experimental series 1, S2, experimental series 2.

Fig. S2: Sample quality assessment *via* MDS. Dimensionality reduction was applied using MDS, in order to visualize the similarities between the expression profiles of the 96 samples and to detect possible outliers and confounding effects. Distances on the plot approximate the typical log2FC between the samples, *i.e.*, one unit represents a FC of 2. The two principal factors distinguishing the expression profiles are personal background and type of treatment.

Fig. S3: Global effects of treatment. MA-plots monitor the effects of treatment with 250 nM vitamin D_3, 250 nM 25(OH)D_3, 10 nM 1,25(OH)$_2D_3$, 100 nM 25(OH)D_3, 1000 nM 25(OH)D_3 and 10,000 nM 25(OH)D_3. The difference in expression change (log2FC) for each tested gene is compared with the mean expression level between the compared groups (log2CPM). Significantly (FDR < 0.05) up- and down-regulated genes are highlighted in red and blue, respectively. The horizonal red lines indicate the borders of absolute FC > 2.

Fig. S4: Individual-specific differential gene expression. Venn diagrams represents the overlap of all target genes of 1000 nM 25(OH)D_3, 10,000 nM 25(OH)D_3 or 10 nM 1,25(OH)$_2D_3$ for individual #05 (**A**), individual #12 (**B**) and individual #14 (**C**).

Fig. S5: Compound-specific differential gene expression. Venn diagrams represent the overlap of target genes of 1000 nM 25(OH)D_3 (**A**) 10,000 nM 25(OH)D_3 (**B**) and 10 nM 1,25(OH)$_2D_3$ (**C**) for individuals #05, #12 and #14.

Fig. S6: Profile of selected vitamin D target genes. The change of expression (log2FC) of the up-regulated vitamin D target genes *TREM1* and *PDPN* as well as of the down-regulated genes *ARHGEF40, LAD1*, *TNFRSF18*, *HCAR3*, *CLEC5A* and *IL13RA1* are based on means of three replicates of individuals #05, #12 and #14.
