# Supplementary figures and images for: Transcriptome-Wide Profile of 25-Hydroxyvitamin D3 in Primary Immune Cells from Human Peripheral Blood
Source: Nutrients. 2021 Nov 16;13(11):4100. doi: 10.3390/nu13114100 (PMC8624141; doi:10.3390/nu13114100)

Fig. S1

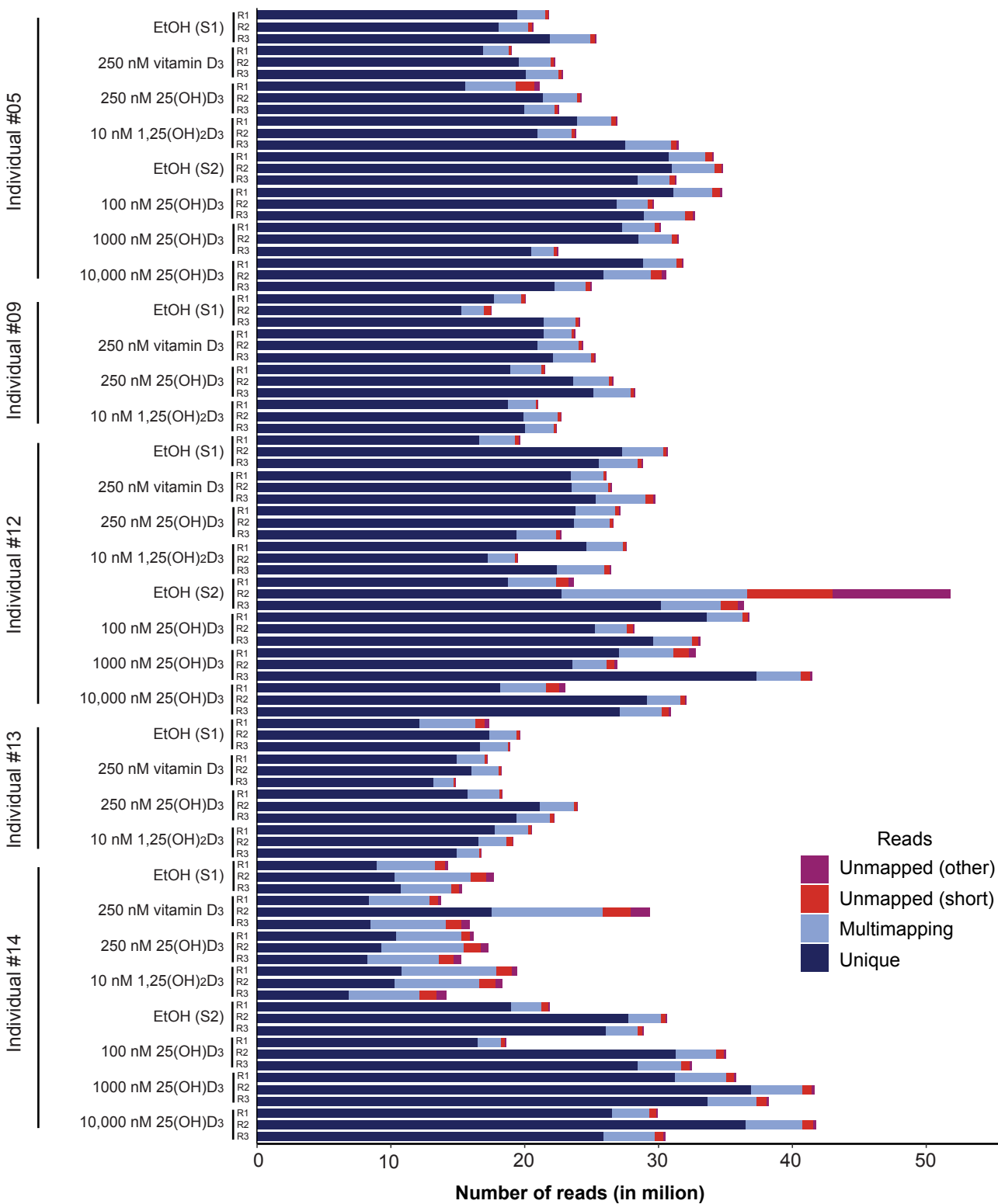

Supplement: Supplementary file 1 [file nutrients-13-04100-s001.zip › nutrients-1405716-supplementary/Supplementary material/Fig S1.pdf]

**Fig. S2**

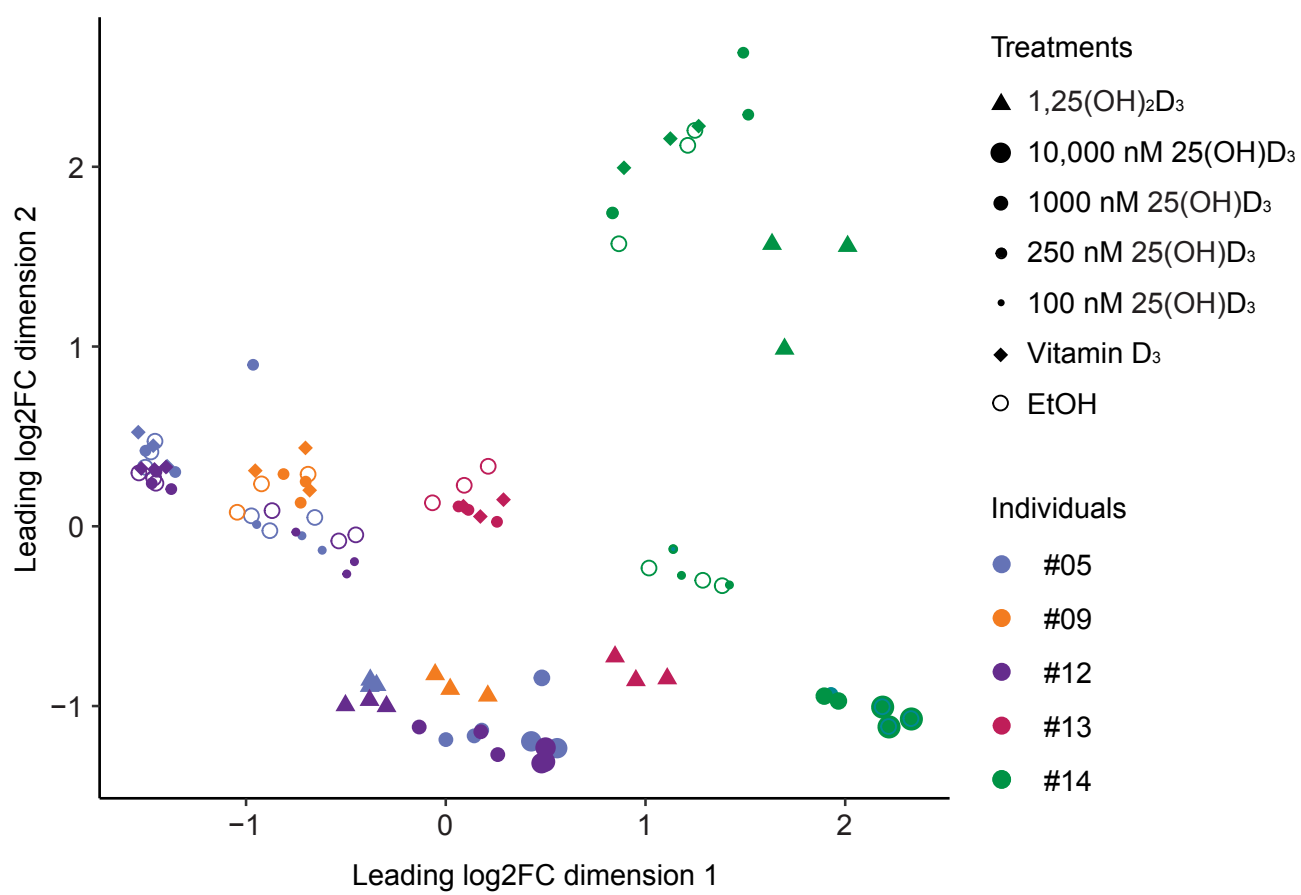

Supplement: Supplementary file 1 [file nutrients-13-04100-s001.zip › nutrients-1405716-supplementary/Supplementary material/Fig S2.pdf]

Fig. S3

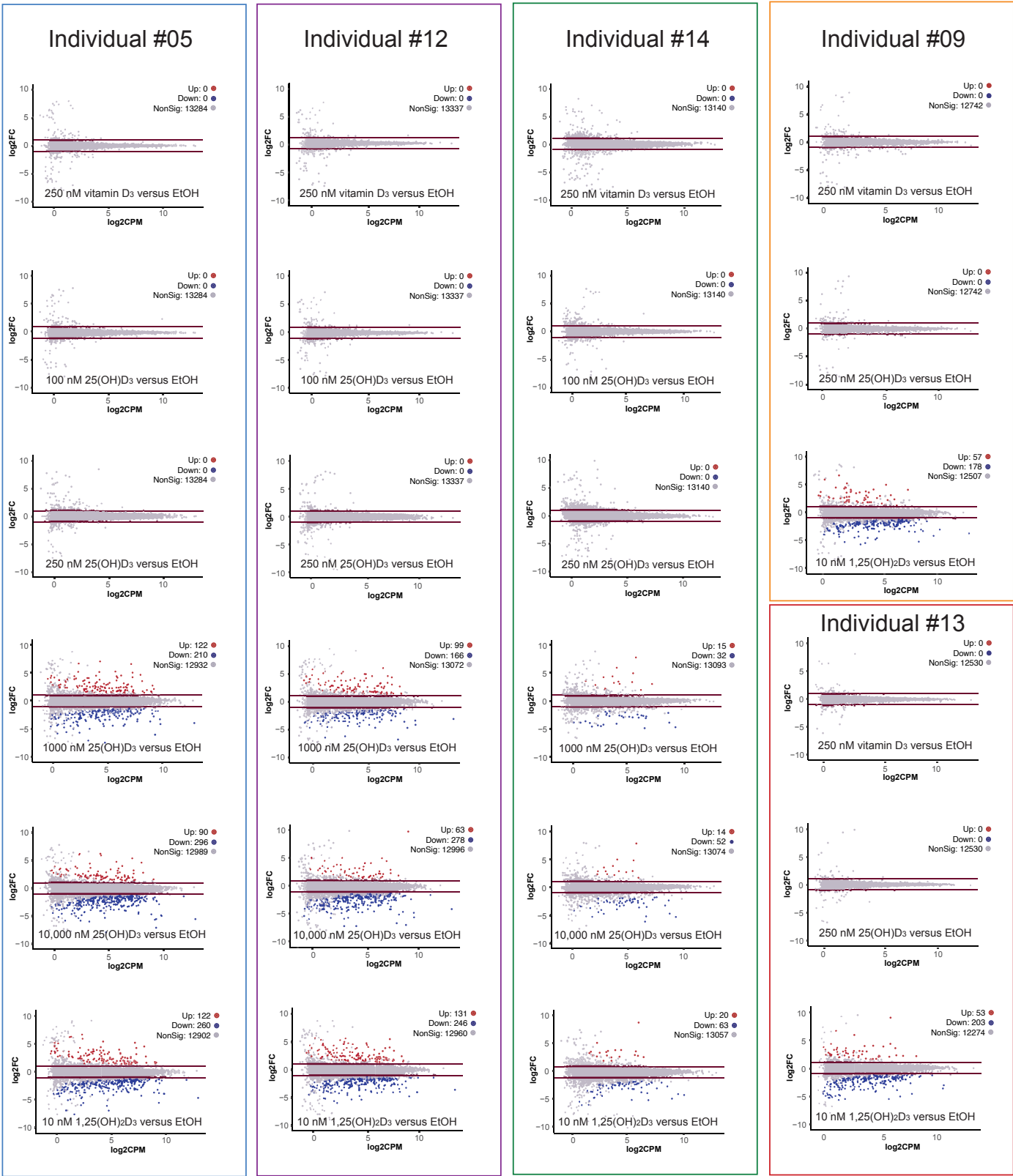

Supplement: Supplementary file 1 [file nutrients-13-04100-s001.zip › nutrients-1405716-supplementary/Supplementary material/Fig S3.pdf]

**Fig. S4**

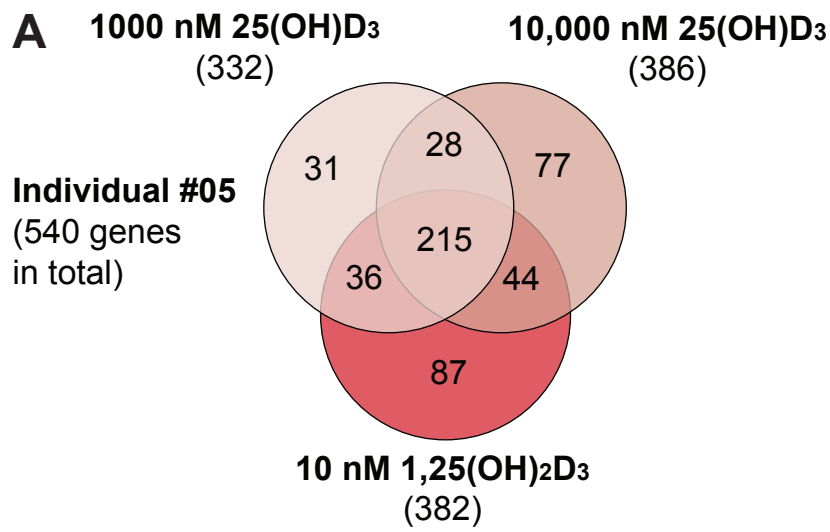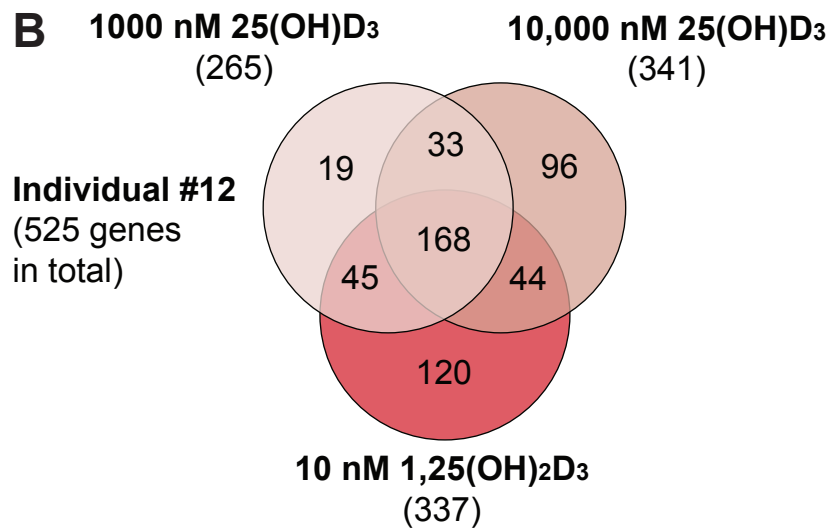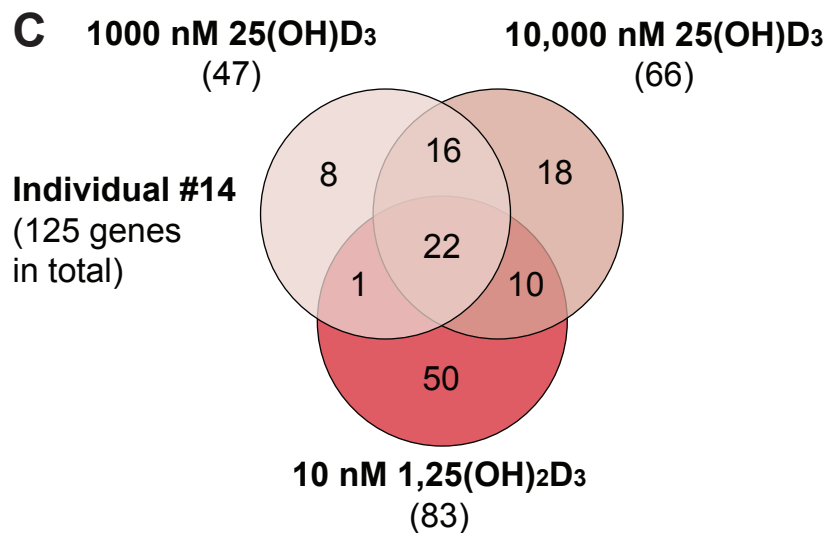

Supplement: Supplementary file 1 [file nutrients-13-04100-s001.zip › nutrients-1405716-supplementary/Supplementary material/Fig S4.pdf]

**Fig. S5**

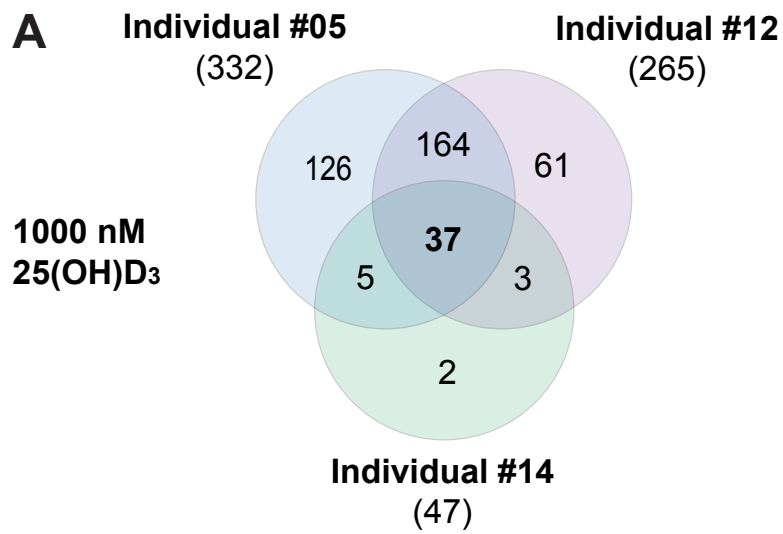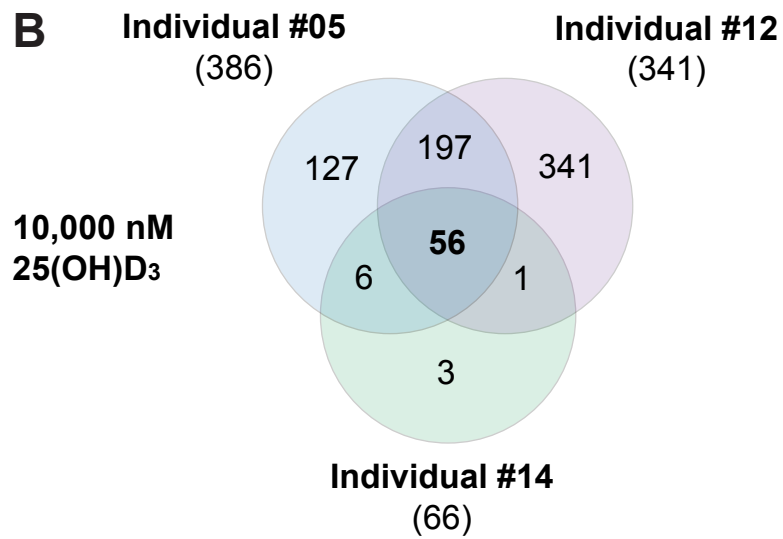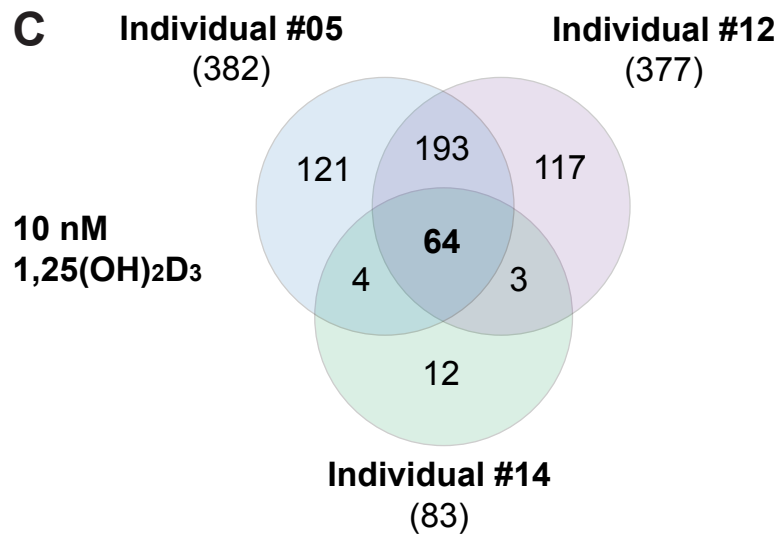

Supplement: Supplementary file 1 [file nutrients-13-04100-s001.zip › nutrients-1405716-supplementary/Supplementary material/Fig S5.pdf]

**Fig. S6**

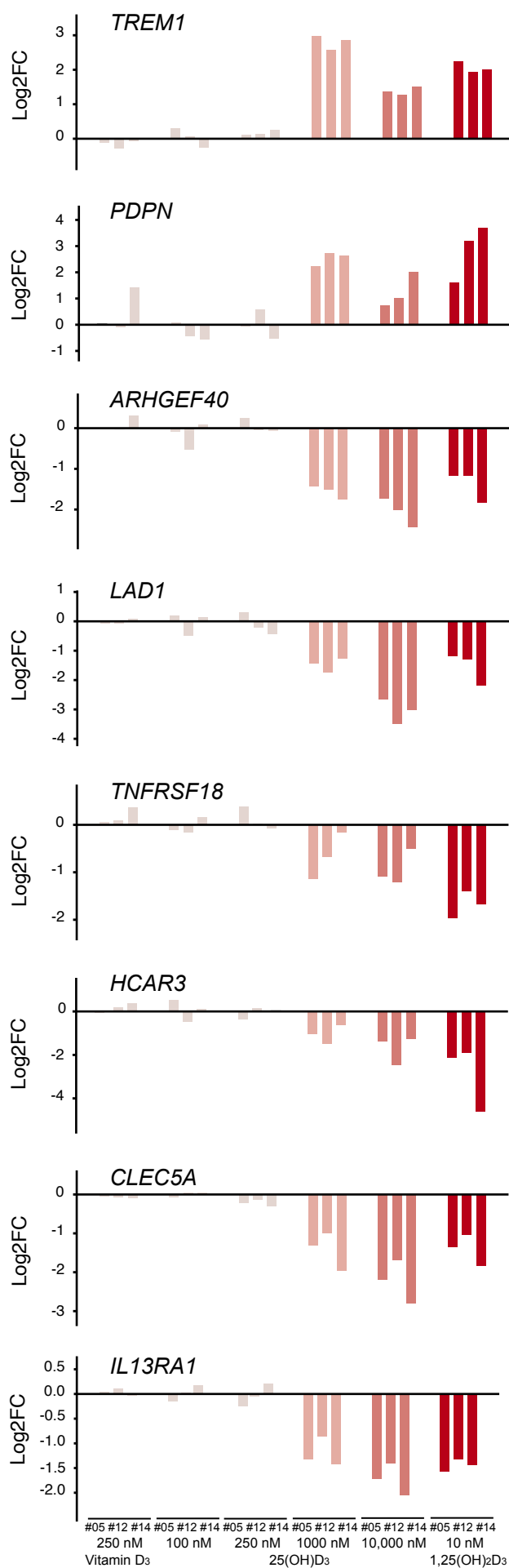

Supplement: Supplementary file 1 [file nutrients-13-04100-s001.zip › nutrients-1405716-supplementary/Supplementary material/Fig S6.pdf]
